# Emergency Medicine Resident Perceptions of Medical Professionalism

**DOI:** 10.5811/westjem.2016.2.29102

**Published:** 2016-05-02

**Authors:** Joshua Jauregui, Medley O. Gatewood, Jonathan S. Ilgen, Caitlin Schaninger, Jared Strote

**Affiliations:** * University of Washington, Department of Emergency Medicine, Seattle, Washington; † University of Cincinnati, Department of Emergency Medicine, Cincinnati, Ohio

## Abstract

**Introduction:**

Medical professionalism is a core competency for emergency medicine (EM) trainees; but defining professionalism remains challenging, leading to difficulties creating objectives and performing assessment. Because professionalism is dynamic, culture-specific, and often taught by modeling, an exploration of trainees’ perceptions can highlight their educational baseline and elucidate the importance they place on general conventional professionalism domains. To this end, our objective was to assess the relative value EM residents place on traditional components of professionalism.

**Methods:**

We performed a cross-sectional, multi-institutional survey of incoming and graduating EM residents at four programs. The survey was developed using the American Board of Internal Medicine’s “Project Professionalism” and the Accreditation Council of Graduate Medical Education definition of professionalism competency. We identified 27 attributes within seven domains: clinical excellence, humanism, accountability, altruism, duty and service, honor and integrity, and respect for others. Residents were asked to rate each attribute on a 10-point scale. We analyzed data to assess variance across attributes as well as differences between residents at different training levels or different institutions.

**Results:**

Of the 114 residents eligible, 100 (88%) completed the survey. The relative value assigned to different professional attributes varied considerably, with those in the altruism domain valued significantly lower and those in the “respect for others” and “honor and integrity” valued significantly higher (p<0.001). Significant differences were found between interns and seniors for five attributes primarily in the “duty and service” domain (p<0.05). Among different residencies, significant differences were found with attributes within the “altruism” and “duty and service” domains (p<0.05).

**Conclusion:**

Residents perceive differences in the relative importance of traditionally defined professional attributes and this may be useful to educators. Explanations for these differences are hypothesized, as are the potential implications for professionalism education. Because teaching professional behavior is taught most effectively via behavior modeling, faculty awareness of resident values and faculty development to address potential gaps may improve professionalism education.

## INTRODUCTION

Medical Professionalism is one of six core competencies required by the Accreditation Council for Graduate Medical Education (ACGME). Emergency medicine (EM) residents must demonstrate a commitment to carrying out professional responsibilities and an adherence to ethical principles.^1^ However, over a decade after the implementation of these standards, the teaching and assessment of professionalism remains a challenge, in great part due to a lack of consensus on its definition.^2^–^6^ One of the greatest challenges to defining professionalism is its dynamic nature: reflecting ever-evolving expectations of patients and physicians in society, particularly regarding attributes that reflect the core of the doctor-patient relationship.

In this context, it is important to understand trainees’ perspectives on what constitutes professionalism. From a top-down viewpoint, these residents’ culture and core values will inform professionalism standards in the future for emergency medicine. From a bottom-up consideration, it is important to measure a baseline set of values to inform and prioritize educational goals. Despite its importance, there have been few studies examining the values that residents place on different aspects of professionalism and none focusing solely on EM trainees.^7^–^10^

The primary objective of this study was to explore current general conceptualizations of professionalism among EM residents by assessing the relative value these trainees place on various professionalism attributes. The secondary objectives were to compare interns’ and seniors’ responses as a proxy of how of clinical and training experiences in EM may shape these values and to compare resident responses across four different sites to explore potential site- or region-specific differences.

## METHODS

### Study Design and Population

This cross-sectional study surveyed a convenience sample of incoming and graduating residents at four EM residency programs representing the South (A), West (B), Midwest (C) and Northeast (D) regions of the United States. In 2011, incoming residents were polled during the first two months of their internship and graduating residents were polled within two months of graduation.

### Survey Instrument

Using the American Board of Internal Medicine’s (ABIM’s) “Project Professionalism” and the ACGME’s definition of professionalism competency as guiding frameworks, we identified seven domains of professionalism (clinical excellence, humanism, accountability, altruism, duty and service, honor and integrity, respect for others) to be represented in the survey. Project Professionalism was a comprehensive multi-year undertaking by the ABIM to provide a modern definition, raise awareness, and guide education and assessment.^11^ Similarly, the ACGME’s definition is a core element of an initiative created jointly with the American Board of Medical Specialties to identify key educational elements of physician competency.^12^

Each domain had several specific attributes and each attribute was represented by an individual item. All items were developed through an iterative process by EM faculty after a review of the literature and published standards of professionalism. Subjects were asked to rate, on a 10-point scale, to what extent each of 27 attributes contributed to their concept of medical professionalism with “none” and “completely” used as anchors at each end of the scale. In addition, subjects were asked whether professionalism was teachable in medical school or residency and whether these attributes could be assessed. We collected additional demographics, including residency location and year of training.

We pilot tested the survey with 10 intern and senior internal medicine and EM residents at a single site for response process and for clarity.^13^ Feedback was incorporated into the final draft, and adaptations of items for the finalized instrument were based upon group consensus of the authors. Both the draft and final instruments had a total of 27 items. The survey instrument is provided in Appendix A.

After the study was completed, in order to assess the internal structure of the instrument, we calculated internal consistency (n=100), using Cronbach’s alpha for the entire 27-item survey and for each of its domains.^14^ These values—representing the degree to which the instrument or domains map to the construct of professionalism or its domains, respectively—were classified a priori as “suboptimal” (values <0.70), “good” (0.70–0.89) or “substantial” (>0.90).^15^ Across the survey in its entirety, internal consistency was substantial (0.91). Within domains, internal consistency was good for clinical excellence (0.75), humanism (0.75), altruism (0.76), duty and service (0.83) and honor and integrity (0.77); internal consistency was suboptimal within the domains of accountability (0.52) and respect for others (0.66).

Also assessed post hoc, in order to examine whether each question added to the survey, we assessed differences in distribution among responses within each domain using Wilcoxon signed rank repeated measures (two items) and Friedman chi-squared test for repeated measures (three or more items). This analysis demonstrated statistically significant differences (p<0.02).

### Survey Protocol

We recruited participants by in-person, phone, and email requests. The survey was administered via an anonymized, secure, web-based platform. All participation was voluntary and there was no compensation for taking the survey. We defined response rate as those who submitted the survey, regardless of the time or form of request they were responding to. The Human Subjects Division at the primary author’s institution study approved the study with a waiver of consent.

### Data Analysis

Data were compiled and entered into SPSS Statistics ver. 22, IBM Corporation (Chicago, IL).

We used descriptive statistics to measure the mean and median for each item. Differences in mean scores for each domain were compared using repeated measures analysis of variance with Bonferroni-corrected post hoc comparisons. We performed two-tailed t-tests for each item, comparing responses from 1) incoming and graduating residents and 2) males and females. A one-way analysis of variance (ANOVA) was used to compare responses from the different residencies. We considered a p-value less than 0.05 statistically significant.

## RESULTS

Of the 114 residents eligible to complete the survey, 100 (88%) completed it, with 36 (100%), 22 (92%), 19 (79%), and 23 (77%) completed at the South, West, Midwest and Northeast residencies respectively. Interns represented 54% of the sample and 55% were male. Males represented 29% of the interns and 84% of the senior residents.

Mean and median scores for each professionalism attribute for both incoming and graduating residents are shown in Table 1. Scores varied considerably, with means ranging from 4.6 to 9.6.

Table 2 shows mean scores within each professionalism domain. A one-way ANOVA revealed a significant difference in the mean domain scores (F=63.3, p=<0.001), which is attributable to lower scores in items related to “altruism” (p=≤0.004, differences ranged from 0.72 to 2.54), and higher scores in “respect for others” (p=0.000, differences ranged from 0.89 to 2.33) as well as “honor and integrity” (p=0.000, differences ranged from 1.1 to 2.54), relative to the other domains. There was no significant difference between “respect for others” and “honor and integrity” (p=0.182).

A significant difference (p<0.05) was found between incoming and graduating residents for five attributes, each corresponding to an individual item under a domain (Table 3). These included “Commitment to lifelong learning,” “I should be an active leader in my community,” “A portion of my care for patients should be for those without pay,” “Active involvement in teaching and/or a professional organization,” and “Compassion and empathy.” In each case, the graduating seniors assigned less value when compared to their incoming intern counterparts.

Differences among residencies were significant for three attributes: “I should always be there for my patients” (p=0.04); “ In an emergency, putting the welfare of others over my own” (p=0.001); and “My patients’ welfare should come above my need for sleep” (p=0.02) (Table 4).

Resident responses to specific professionalism teachability/testability questions are summarized in Table 5. Overall, 82% felt that professionalism was teachable to residents, but only 37% thought it could be assessed.

## DISCUSSION

Our assessment of EM residents’ self-reported conception of professionalism revealed variance in value placed on the different domains of professionalism competency as defined by the ACGME. This study adds to a growing literature on resident perspectives^7^–^10^ and is the first to focus solely on EM residents.

Given the unique training experiences, cultural environments, and work practices of each specialty, different concepts of professionalism may be emphasized; analyses between residents of different specialties have confirmed such differences.^8^ Prior studies that pooled residents from multiple disciplines may therefore demonstrate additional variability or diluted results that reflect these different populations, making their results less generalizable to EM trainees.

Our study differs from others in an additional way. The majority of prior studies were performed in a structured interview or focus group format,^7^–^10^ which risk an “interview effect:” values that could be perceived as different from traditional norms (such as minimizing the placement of others before oneself) could be de-emphasized. Our anonymous survey allowed residents to clearly and safely appraise concepts of professionalism without risk of judgment.

Our most notable finding, and different from prior studies, was that “altruism” was rated significantly lower than all other domains. Altruism can be defined as when a physician, “adheres to (the) best interest of the patient; (and) puts (the) best interest of the patient above self-interest and the interest of other parties.”^16^ As the ACGME states in its common program requirements, “Residents are expected to demonstrate responsiveness to patient needs that supersede self-interest.”^17^

It is likely that our residents’ responses reflect a current reconceptualization of the traditional concept of altruism. Wellness, including restrictions on patient care such as work-hour limits, has been a priority for the entirety of their medical training, creating a culture where higher value may be frequently placed on physician self-interest than patient needs.^20^ The increasing ACGME emphasis on wellness both reflects and drives this change.

It should be noted that physician altruism is a complicated concept with widely varying interpretations.^18^ One prior study found that residents perceived a focus on work-life balance to enhance professionalism by promoting well-being and teamwork;^21^ others note a perceived conflict between altruism and self-interest.^22^ A common concern in the literature regarding the development of professionalism in our learners is the continual commercialization of medicine and ongoing evolution of the biological and technical aspects of practice.^2^ As physicians become regarded more as service “providers,” an unintended consequence may be that our millennial learners are becoming less “patient relationship-focused” and more “commodity-focused” learners.^23^

Although the effects of changes in the perceived role of altruism as part of medical professionalism remain unclear, the speed of change in this domain relative to others likely creates an increasingly difficult divide between educators and learners in teaching and assessment. There is evidence that professionalism is largely learned in an implicit and experiential manner, creating difficulties for both faculty development and role-modeling when significant differences in values exist.^28^

If, as a specialty, we are committed to creating physicians who place high value on all traditional professional concepts, the path may be difficult. In our study, for example, the values of many attributes were rated significantly lower when evaluated in more experienced residents. Devaluing “commitment to lifelong learning” is notable, given their recent immersion in focused learning and the early point in their educational journey. Lowering the value placed on “a portion of my care for patients should be for those without means to pay” seems to be misaligned with EM’s commitment to being the safety net for a community’s healthcare needs. The decreased significance of “compassion and empathy” also seems out of sync with EM’s core values, and may reflect changes in role-modeling or organizational priorities in our teaching hospitals, or a natural cynicism arising from experiences in patient care.

Although there is no way to fully assess the multi-factorial causes of these changes we found, some of the differences seen are consistent with studies of medical students that show a similar progressive decrease in baseline humanistic and empathic qualities.^24^–^27^ Such changes have been postulated to be due at least in part to an informal curriculum (interpersonal experiences and work expectations) that devalues altruism as well as a hidden curriculum (organizational structure and culture) in academic medical centers that may place value on metrics such as efficiency or billing over altruism.^28^–^30^

Although it is clear to the teachers that professionalism is difficult to teach, our participants overwhelmingly believe that it can be taught effectively. And while not formally analyzed, comments from the residents entered as free text in the survey consistently agreed that role modeling was the best way for them to learn professionalism. In a recent “Best Evidence in Medical Education” review, role modeling and mentoring were considered to be the most effective techniques for developing professionalism.^24^ And when EM and surgical residents were asked about their perspectives on professionalism, learning professionalism through role modeling was the most common theme.^7^,^31^ Despite these findings, fewer than half of U.S. and Canadian medical schools report providing formal faculty development in mentoring and only 8% provide assistance in the development and nurturing of professionalism.^32^ In addition, role models are often unaware of their educational impact, making faculty development or a reliance on informal teaching a challenge.^29^ Improved and increased faculty development, therefore, may be the low-hanging fruit to improve our ability to reinforce and teach professional values in our residents.

Such development may need to take into account local custom and culture if there are, in fact, differences at training sites. Although survey responses may not adequately assess actual ethical and cultural values, among the four different institutions included in our study, residents’ self-conception of professionalism differed significantly among three attributes, two in the altruism category and one in the duty category. Of note, one of the institutions studied was a military emergency medicine residency program where much of the statistical difference occurred. These physicians’ professional values as military officers may have impacted their responses to the different professional attributes,^33^ reflecting how social pressures and environmental constraints influence professional attitudes and behaviors.^10^ The power of institutional culture on individual learning of professionalism is significant and can also potentially inform education and evaluation reform.^34^

One final challenge to professionalism education is assessment, viewed pessimistically by the majority of our respondents. Consistent with that sentiment, a 2012 consensus conference working group on assessing professionalism in EM concluded that existing instruments demonstrate insufficient reliability and validity to provide psychometrically robust assessment.^4^ Because professionalism is a “complex construct” this group recommended that it be evaluated with multiple methods, including personal portfolios and narratives, simulation, and direct observation among others.^4^ A significant challenge in assessing professionalism is adequate faculty awareness and confidence,^35^ and further emphasis on teacher training could potentially provide substantial benefits in this regard as well.

## LIMITATIONS

There are several limitations to our study. Only 86% of residents responded; those who did not respond may have represented a different population that could substantially change our findings. Furthermore, variability of response rate from different institutions could have skewed the differences among institutions. Areas where no difference was found should be viewed with caution, as the study was designed to be primarily descriptive in nature and was not prospectively powered. Furthermore, the study results are largely descriptive and limited by the methods in its ability to draw any comparative conclusions to current professionalism conceptualizations. As this was the first use of this survey instrument, its validity evidence is thus limited, and there is potential that scores do not adequately reflect resident perceptions of professionalism.

Given the large number of items included in the survey, it is possible that significant differences exist purely due to sampling error. In addition, the domains of professionalism in our survey instrument may be interdependent, but we did not test this.

Also, we attempted to study a representative sample of the population (EM residents) of interest by surveying residents from four different institutions from four different regions of the country; however, this extremely limited sample (including a military residency that may differ from non-military residencies in critical ways) may not properly reflect EM residencies as a whole. Although we found certain differences between residency programs this cannot be interpreted as differences between regions.

When comparing interns and graduating residents, we only looked at one snapshot of time. We also did not compare individuals before and after training or examine personal, educational, or cultural factors that could have had influenced changes. We present the data for consideration only and do not attempt to draw conclusions about the effect of experience or training. Each residency had professionalism training and assessment during the period in question that may have affected the results. None of these were formal programs and given the ambiguity surrounding them, we chose not to include them in the analysis.

Given all these limitations, the data in this study are provided to guide further research and education programs, rather than to draw definitive conclusions.

## CONCLUSION

The relative value assigned to different professional attributes by the residents we surveyed showed variance and was significantly lower in the altruism domain. Differences were also found comparing learners at different levels of training and location. These findings likely reflect, at least in part, multiple different challenges in defining and teaching professionalism. Because the concept of professionalism is dynamic and likely best taught through role-modeling, increased faculty development, including an understanding of the current generations’ perceptions, should probably play a significant role in professionalism education.

## Supplementary Information



## Figures and Tables

**Table 1:** Value placed by residents on each medical professionalism attribute.

Domain	To what extent do the following contribute to your concept of medical professionalism?	Mean	95% CI (Mean)	Median
Excellence	Excellence in communication and listening	9.0	8.8–9.3	9
	Technical competence, skill, excellence	8.6	8.3–9.0	9
	Hard work and discipline	8.7	8.4–9.0	9
	Ability to make difficult decisions with limited information	7.5	7.0–8.0	8
	Commitment to lifelong learning	8.7	8.4–9.0	9
Humanism	Compassion and empathy	8.9	8.6–9.2	9
	Emotional Intelligence	8.4	8.0–8.7	9
	An artist as much as a scientist	6.7	6.3–7.2	7
	Commitment to social justice	7.5	7.1–7.9	8
Accountability	Self-reflection and insight	8.1	7.7–8.4	8
	Taking responsibility for mistakes	9.3	9.0–9.5	10
	Autonomy in my decision making	7.8	7.4–8.2	8
Altruism	My patients’ welfare should come before my need for balance in my life	5.1	4.6–5.6	5
	My patients’ welfare should come above my financial interests	8.6	8.3–8.9	9
	My patients’ welfare should come above my need for sleep	4.6	4.1–5.1	4
	In an emergency, putting the welfare of others over my own safety	4.8	4.2–5.3	5
Duty and service	I should always be there for my patients	8.1	7.8–8.5	8
	I should be an active leader in my community	7.6	7.3–8.0	8
	Active involvement in teaching and/or a professional organization	7.6	7.2–8.0	8
	A portion of my care for patients should be for those without means to pay	7.6	7.2–8.0	8
	I should volunteer my skill and expertise for the welfare of the community	7.5	7.1–7.9	8
Honor and integrity	Honesty	9.6	9.5–9.8	10
	Commitment to one’s personal and professional codes	9.1	8.9–9.4	10
	My behavior should be used as a model for the community	7.9	7.5–8.3	8
	My behavior away from work should be respectable	8.5	8.2–8.8	9
Respect for others	All patients should be treated equally	8.9	8.6–9.2	10
	Respect for co-workers	9.4	9.2–9.6	10

**Table 2:** Mean resident responses for each domain of professionalism.

Domain	Mean (SD)
Excellence	8.27 (2.02)
Humanism	7.56 (2.20)
Accountability	7.93 (1.94)
Altruism	6.83 (2.69)
Duty and service	7.79 (1.95)
Honor and integrity	9.37 (1.09)
Respect for others	9.16 (1.32)

Note: Significance of mean domain differences: Differences in mean scores for each domain was compared using repeated measures analysis of variance with Bonferroni-corrected post hoc comparisons. F=63.3, P=<0.001. Altruism < all others; Respect for others, Honor and integrity > all others.

**Table 3:** Difference between intern and senior resident responses.

Attribute	Incoming residents’ mean score (SD)	Graduating residents’ mean score (SD)	P value^a^
Commitment to lifelong learning	9.02 (1.35)	8.39 (1.51)	0.031
I should be an active leader in my community	8.00 (1.79)	7.22 (1.99)	0.042
A portion of my care for patients should be for those without means to pay	8.04 (1.88)	7.13 (2.20)	0.029
Active involvement in teaching and/or a professional organization	8.11 (1.72)	7.00 (1.96)	0.003
Compassion and empathy	9.17 (1.11)	8.54 (1.93)	0.047

a Comparing mean scores between incoming and graduating residents using a two-tailed t test.

**Table 4:** Differences among residency programs.

Attribute	South (SD)	West (SD)	Midwest (SD)	Northeast (SD)	P value^a^
I should always be there for my patients	7.78 (1.72)	8.82 (1.18)	7.47 (1.87)	8.50 (2.13)	0.042 F[3,96] = 2.83
In an emergency, putting the welfare of others over my own	4.14 (2.29)	6.41 (3.16)	5.00 (2.47)	4.05 (2.44)	0.006 F[3,96] = 4.37
My patients’ welfare should come above my need for sleep	4.43 (2.14)	5.77 (2.96)	3.37 (2.31)	4.77 (2.37)	0.02 F[3,96] =3.45

a Comparing attribute scores among four different residencies using one-way ANOVA.

F, F statistic. Number of responses=100.

**Table 5:** Resident responses to specific professionalism questions.

Professionalism questions	Yes	No
Is professionalism teachable through a residency curriculum?	82%	18%
Is professionalism testable?	37%	63%
