# Supplementary material for: Emergency Medicine Resident Perceptions of Medical Professionalism
Source: West J Emerg Med. 2016 May 2;17(3):355–61. doi: 10.5811/westjem.2016.2.29102 (PMC4899070; doi:10.5811/westjem.2016.2.29102)
Supplement: Supplementary file 1 [file wjem-17-355-s001.pdf]

Appendix A: The Survey Instrument

Medical Professionalism Survey

	**To what extent do each of the following contribute to your concept of Medical Professionalism?**	**None**									**Completely**
1.	Excellence in communication and listening	1	2	3	4	5	6	7	8	9	10
2.	All patients should be treated equally	1	2	3	4	5	6	7	8	9	10
3.	I should always be there for my patients	1	2	3	4	5	6	7	8	9	10
4.	Ability to make difficult decisions with limited information	1	2	3	4	5	6	7	8	9	10
5.	Commitment to lifelong learning	1	2	3	4	5	6	7	8	9	10
6.	My patients' welfare should come above my financial interests	1	2	3	4	5	6	7	8	9	10
7.	Emotional intelligence	1	2	3	4	5	6	7	8	9	10
8.	An artist as much as a scientist	1	2	3	4	5	6	7	8	9	10
9.	Self-reflection and insight	1	2	3	4	5	6	7	8	9	10
10.	I should volunteer my skill and expertise for the welfare of the community	1	2	3	4	5	6	7	8	9	10
11.	Commitment to social justice	1	2	3	4	5	6	7	8	9	10
12.	Autonomy in my decision making	1	2	3	4	5	6	7	8	9	10
13.	My patients' welfare should come before my need for balance in my life	1	2	3	4	5	6	7	8	9	10
14.	In an emergency, putting the welfare of others over my own safety	1	2	3	4	5	6	7	8	9	10
15.	Hard work and discipline	1	2	3	4	5	6	7	8	9	10
16.	I should be an active leader in my community	1	2	3	4	5	6	7	8	9	10
17.	A portion of my care for patients should be for those without means to pay	1	2	3	4	5	6	7	8	9	10
18.	Honesty	1	2	3	4	5	6	7	8	9	10
19.	Active involvement in teaching and/or a professional organization	1	2	3	4	5	6	7	8	9	10
20.	Compassion and empathy	1	2	3	4	5	6	7	8	9	10
21.	My patients' welfare should come above my need for sleep	1	2	3	4	5	6	7	8	9	10
22.	Taking responsibility for mistakes	1	2	3	4	5	6	7	8	9	10
23.	Commitment to one's personal and professional codes	1	2	3	4	5	6	7	8	9	10
24.	My behavior should be used as a model for the community	1	2	3	4	5	6	7	8	9	10
25.	Technical competence, skill, excellence	1	2	3	4	5	6	7	8	9	10
26.	My behavior away from work should be respectable	1	2	3	4	5	6	7	8	9	10
27.	Respect for co-workers	1	2	3	4	5	6	7	8	9	10

28. Is professionalism teachable through a residency curriculum? Y N

    Comments on why or why not:

29. Is professionalism testable? Y N

    Comments on why or why not:
